# Process Optimization of Ultra-High Molecular Weight Polyethylene/Cellulose Nanofiber Bionanocomposites in Triple Screw Kneading Extruder by Response Surface Methodology

**DOI:** 10.3390/molecules25194498

**Published:** 2020-09-30

**Authors:** Nur Sharmila Sharip, Hidayah Ariffin, Yoshito Andou, Yuki Shirosaki, Ezyana Kamal Bahrin, Mohammad Jawaid, Paridah Md Tahir, Nor Azowa Ibrahim

**Affiliations:** 1 Institute of Tropical Forestry and Forest Products (INTROP), Universiti Putra Malaysia, Serdang 43400, Selangor, Malaysia; nursharmilasharip@gmail.com (N.S.S.); jawaid@upm.edu.my (M.J.); parida.introp@gmail.com (P.M.T.); 2 Department of Bioprocess Technology, Faculty of Biotechnology and Biomolecular Sciences, Universiti Putra Malaysia, Serdang 43400, Selangor, Malaysia; ezyana@upm.edu.my; 3 Department of Biological Functions and Engineering, Kyushu Institute of Technology, Graduate School of Life Science and Systems Engineering, 2-4 Hibikino, Wakamatsu-ku, Kitakyushu, Fukuoka 808-0196, Japan; yando@life.kyutech.ac.jp; 4 Department of Applied Chemistry, Kyushu Institute of Technology, Faculty of Engineering, 1-1 Sensui-cho, Tobata-ku, Kitakyushu, Fukuoka 804-8550, Japan; yukis@che.kyutech.ac.jp; 5 Department of Chemistry, Faculty of Sciences, Universiti Putra Malaysia, Serdang 43400, Selangor, Malaysia; norazowa@upm.edu.my

**Keywords:** ultra-high molecular weight polyethylene, cellulose nanofiber, bionanocomposite, response surface methodology, optimization, melt-blend processing

## Abstract

Incorporation of nanocellulose could improve wear resistance of ultra-high molecular weight polyethylene (UHMWPE) for an artificial joint application. Yet, the extremely high melt viscosity of the polymer may constrict the mixing, leading to fillers agglomeration and poor mechanical properties. This study optimized the processing condition of UHMWPE/cellulose nanofiber (CNF) bionanocomposite fabrication in triple screw kneading extruder by using response surface methodology (RSM). The effect of the process parameters—temperature (150–190 °C), rotational speed (30–60 rpm), and mixing time (30–45 min)—on mechanical properties of the bionanocomposites was investigated. Homogenous filler distribution, as confirmed by scanning electron microscopy-energy dispersive spectroscopy (SEM-EDS) analysis, was obtained through the optimal processing condition of 150 °C, 60 rpm, and 45 min. The UHMWPE/CNF bionanocomposites exhibited improved mechanical properties in terms of Young’s and flexural modulus by 11% and 19%, respectively, as compared to neat UHMWPE. An insignificant effect was observed when maleic anhydride-*grafted*-polyethylene (MAPE) was added as compatibilizer. The obtained results proved that homogenous compounding of high melt viscosity UHMWPE with CNF was feasible by optimizing the melt blending processing condition in triple screw kneading extruder, which resulted in improved stiffness, a contributing factor for wear resistance.

## 1. Introduction

Cellulose nanofibers (CNF) are bio-based, sustainable, and environmentally friendly materials with promising applications as composites filler [1,2,3,4]. Possessing a greater degree of Young’s modulus and tensile strength (up to 180 GPa and 22 Gpa, respectively), CNF enables an increment in mechanical properties, particularly the stiffness of most polymers [5]. Remarkable improvement of polymer properties, such as polylactic acid (PLA), polypropylene (PP), low-density polyethylene (LDPE), high-density polyethylene (HDPE), and polyvinyl alcohol (PVA), has been reported by the reinforcement of less than 10% CNF propitious to various applications in packaging, electronic, building materials, automobile, digital display, pharmaceutical, and biomedical [6,7,8,9,10,11,12,13,14,15].

As such, CNF could be a promising reinforcing agent in enhancing the properties of ultra-high molecular weight polyethylene (UHMWPE). While UHMWPE has been an excellent material choice as an artificial joint component for more than 50 years, it is being used as a polymer on the metal prosthetic joint, causing it to experience wear and deformation under sliding conditions due to relatively low hardness, Young’s modulus, or stiffness as compared to the sliding metal femoral counterpart [16]. According to Zhou et al. [17], the presence of a filler in the UHMWPE matrix may contribute to some load-bearing capacity, such as increased stiffness and hardness, thus reducing the stress applied on the polymer. Studies by Li et al. [18] and Wang et al. [19] showed relatively lower wear volume, which was generated by the incorporation of cellulose nanocrystals (CNC), indicating the potential of nanocellulose materials as a UHMWPE filler for improving the wear resistance. The viability of MC3T3-E1 preosteoblast cells was significantly higher, while the inflammatory response of macrophage RAW 264.7 cells was lower when grown on UHMWPE/CNC composite, proving the biocompatibility and non-toxicity of nanocellulose materials as a UHMWPE filler.

Though, in comparison to other well-studied UHMWPE fillers, including carbon nanofibers, carbon nanotube, and graphene, the uses of nanocellulose, including CNF in the UHMWPE matrix, is much less and yet to be widely studied. This is probably due to the high melt viscosity of UHMWPE, attributed to molecular weights ranging from 3 to 6 million g/mol [20,21], which make dispersing hydrophilic CNF and avoiding filler agglomeration to be very challenging. However, the use of high-speed processing can improve the filler dispersion in the matrix through the generation of high-shear force and high shearing rate [22], notwithstanding associated thermal degradation and/or chain scission of the polymer [23]. Likewise, increasing melt mixing time beyond critical duration can contribute to a satisfactory filler dispersion, yet too long exposure to high temperature could degrade the polymer [24]. Thus, the optimization of the screw rotational speed, temperature, and mixing time is essential to prevent detrimental impacts on the properties of the polymer. In doing so, the use of response surface methodology (RSM) is preferable and promising as it allows the calculation of complex interactions between independent variables, besides the removal of systematic errors and reduction in the number of experiments [25,26]. For instant, adoption of face-centered central composite design, the process optimization of cellulose nanofiber has been widely reported [27,28,29]. Aside from processing parameters, it is also worth noting that the utilization of compatibilizer, such as maleic anhydride-grafted-polyethylene (MAPE), is effective in enhancing filler-matrix interaction in composites [30,31]. Nevertheless, the incorporation of MAPE in UHMWPE blends for improving the rheological properties of the polymer, exhibiting a reverse effect on the mechanical properties of the polymer [32].

In this light of the literature review, cellulose-based material can be an interesting material as a nanofiller in UHMWPE composite due to its biocompatibility and non-toxic property. However, there is not much information available in the literature on the use of CNF as a filler in UHMWPE. Therefore, the effects of processing conditions (temperature, rotational speed, and mixing time) on the filler’s dispersion and mechanical properties were evaluated. Mathematical models between process parameters and responses were generated through the implementation of Design-Expert software. The optimum UHMWPE/CNF bionanocomposite processing condition was acquired, and the validation experiment was carried out to confirm whether the obtained optimized condition leads to desired mechanical properties and homogenous CNF dispersion or not. Herein, the effect of compatibilizer was also investigated.

## 2. Results and Discussion

### 2.1. Analysis of the Model

The tensile strength, yield strength, elongation at break, and Young’s modulus data used in the design matrix generated by Design-Expert software allowed regression analysis to be carried out. This was in order to obtain the best-fit model for the experimental data, whereby the derived regression equation could be used to predict a particular response at points that were not included in the regression. The regression analysis of the experimental data suggested the relationship between temperature, rotational speed, and mixing time as variables, with tensile strength, yield strength, elongation, and Young’s modulus as responses. The experimental and predicted values are shown in Table 1.

Full quadratic models were adopted for all responses as the best-fitted model, as shown in Table 2. Considering a high ratio of maximum to minimum response value of elongation, which was 8.8793 (more than 3), natural log transformation was applied for elongation, as suggested by the software. The transformation of the model was used in a case where residual analysis indicated some problems with the assumed model, such as non-normality or non-constant variance in the responses [33]. Selection of the models was based on the analysis of variance (ANOVA) with significant model probability (*p* < 0.05), insignificant lack-of-fit probability (*p* > 0.005), and satisfactory coefficient of determination (R^2^) (above 80%) [28,34].

Significant *p*-value and insignificant lack-of-fit indicate a good model and a good fit of the model to the data, respectively [35]. In this experiment, the *p*-values for the lack-of-fit test of tensile strength, yield strength, elongation, and Young’s modulus were 0.2230, 0.7311, 0.0726, and 0.7601, respectively (Table 2). These values were higher than 0.05, demonstrating the model had insignificant lack-of-fit. For instance, if the model has a significant lack-of-fit, it should not be used for the prediction of a particular response due to the model’s failure to represent data at points that were not included in the regression.

**Figure 1:**
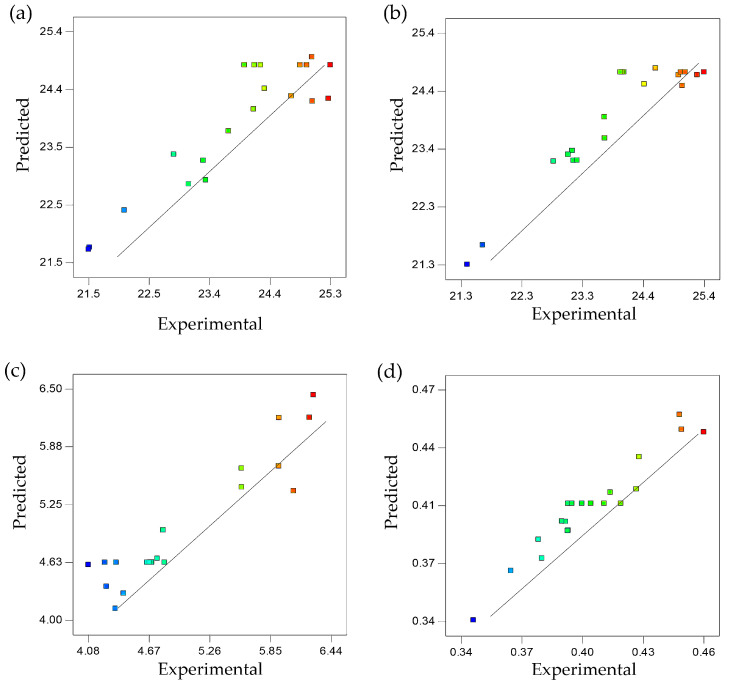
Experimental and predicted values for (**a**) tensile strength, (**b**) yield strength, (**c**) elongation, and (**d**) Young’s modulus of ultra-high molecular weight polyethylene/cellulose nanofibers (UHMWPE/CNF) bionanocomposites.

The coefficient of determination, R^2^, which was close to 1, indicated that the dependent variable was predicted with less error compared to independent variables of temperature, rotational speed, and mixing time. The R^2^ values of 0.8314, 0.8685, 0.8745, and 0.9258, respectively, for all responses, proved that the variance proportion of 83%, 87%, 87%, and 93% in tensile strength, yield strength, elongation at break, and young’s modulus was predictable from temperature, rotational speed, and mixing time of UHMWPE/CNF bionanocomposite fabrication (Table 2). Additionally, an R^2^ value close to 1 indicated good agreement between experimental and predicted values of responses [33,34]. Based on plots in Figure 1, the proximity of points scattered along the fitted line proved agreement between experimental and predicted values, thus confirming the adequacy of models to predict mechanical properties of UHMWPE/CNF bionanocomposites fabricated at different temperatures, rotational speeds, and mixing times. The regression equations to predict the effect of factors on the responses are shown in Equations (1)–(4), where Y_1_, Y_2_, Y_3_, and Y_4_ represent tensile strength, yield strength, elongation at break, and Young’s modulus, respectively; X_1_, X_2_, and X_3_ are mixing temperature, speed, and duration, respectively.

Y1 = 24.84 + 0.71 X_1_ + 0.084 X_2_ + 0.37 X_3_ − 1.23 X_1_^2^ − 0.48 X_2_^2^ − 0.24 X_3_^2^ − 0.017 X_1_X_2_ − 0.058 X_1_X_3_ − 0.24 X_2_X_3_ (1)

Y_2_ = 24.71 + 0.76 X_1_ + 0.059 X_2_ + 0.61 X_3_ − 0.81 X_1_^2^ + 0.00931X_2_^2^ − 0.85 X_3_^2^ + 0.14 X_1_X_2_ − 0.22 X_1_X_3_ + 0.09 X_2_X_3_ (2)

Ln (Y_3_) = 4.62 − 0.65 X_1_ + 0.02 X_2_ − 0.4 X_3_ + 0.39 X_1_^2^ + 0.021 X_2_^2^ + 0.37 X_3_^2^ − 0.23 X_1_X_2_ − 0.15 X_1_X_3_ + 0.13 X_2_X_3_ (3)

Y_4_ = 0.41 + 0.03 X_1_ + 0.009017 X_2_ + 0.019 X_3_ + 0.009901 X_1_^2^ − 0.0009211 X_2_^2^ − 0.012 X_3_^2^ + 0.002125 X_1_X_2_ + 0.000311 X_1_X_3_ − 0.00699 X_2_X_3_ (4)

### 2.2. Effect of Melt-Blending Processing Condition on CNF Dispersion and Mechanical Properties

**Figure 2:**
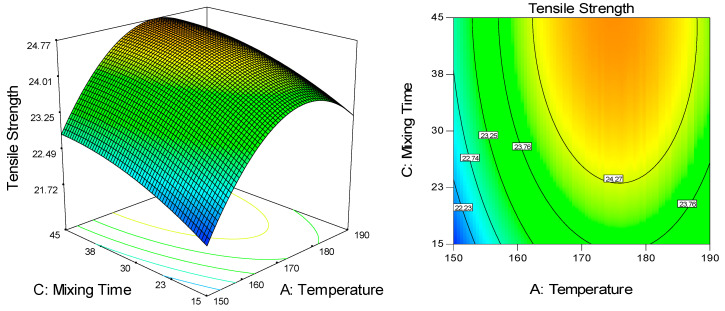
The 3D and contour plot for the dependence of UHMWPE/CNF bionanocomposite’s tensile strength on temperature and mixing time as significant factors.

The effect of each processing factor—temperature, rotational speed, and mixing time—on the mechanical properties of UHMWPE/CNF bionanocomposite was evaluated through the regression analysis of the experimental conditions, according to the quadratic model. Figure 2 illustrates the three-dimensional and contour plot of the response surface for the effects of processing parameters on tensile strength, based on Equation (1). The factors of rotational speed and mixing time were found insignificant, whereas only linear and quadratic effects of temperature were significant (Table 2). The tensile strength of UHMWPE/CNF bionanocomposites was increased by increasing temperature from 150 °C up to 170 °C, before decreasing at a higher temperature of 170 °C to 190 °C. The results obtained herewith suggested an improvement in UHMWPE chain mobility at higher temperature processing, enhanced chain entanglement hence leads to better tensile properties [36,37].

**Figure 3:**
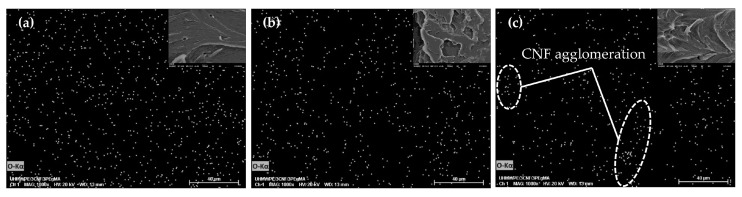
SEM-EDS images of UHMWPE/CNF bionanocomposites fabricated at temperatures (**a**) 150 °C, (**b**) 170 °C, and (**c**) 190 °C at constant rotational speed (45 rpm) and mixing time (30 min).

Nevertheless, the use of high temperature (190 °C) led to CNF agglomeration, as evidenced through SEM-EDS analysis, as shown in Figure 3c. The accumulation of white dots, representing oxygen element which uniquely belongs to CNF in the SEM-EDS images, indicated the presence of CNF agglomeration within the polymer matrix [31,38,39]. At temperatures 150 °C and 170 °C, the CNF was seen to be well distributed without the presence of agglomeration (Figure 3a,b).

**Figure 4:**
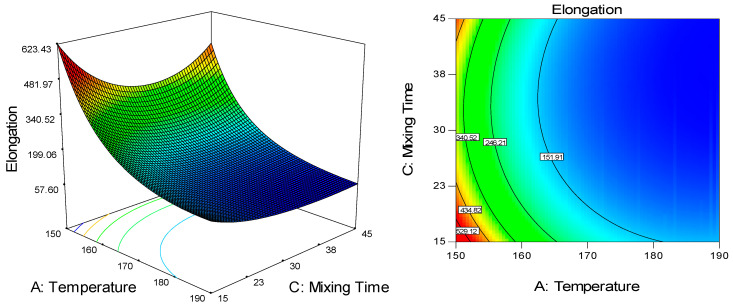
The 3D and contour plot for the dependence of UHMWPE/CNF bionanocomposite’s elongation at break on temperature and mixing time as significant factors.

The significant effect of temperature (linear and quadratic) was also obtained against elongation at break, along with the linear effect of mixing time. The value of elongation at break decreased by increasing temperature and mixing time (Figure 4). However, the effect of mixing time was less prominent in comparison to the effect of temperature by which the coefficient of each factor was 0.40 and 0.65, respectively (Equation (3)). The reduction at an increasing temperature from 150 °C to 190 °C and constant speed and mixing time of 45 rpm and 30 min, respectively, was approximately as much as 81%. This was stemmed from the appearance of large CNF agglomeration at the highest temperature of 190 °C (Figure 3c), as mentioned in the previous paragraph. Higher temperatures can cause faster water vaporization in CNF, leading to agglomeration [40]. CNF suspension fed into the triple screw kneading extruder experienced drying during mixing with UHMWPE by which water evaporation caused capillary forces to pull adjacent fibers together, causing fiber agglomeration. At faster water vaporization, more agglomeration formed due to the inherent tendency of nanofibers forming agglomerates that are attributed by the strong fiber–fiber hydrogen bonding coupled with their polar nature, especially when surrounded by a non-polar polymeric environment [3,40,41].

The presence of agglomeration restricted interfacial properties between the polymer matrix and filler, thus inhibiting the efficient stress transfer when the load was applied. The resulted inefficient stress transfer caused uneven stress distribution, eventually leading to crack or debonding [31,42]. In the event of increased applied load, uneven stress distribution or high-stress concentration onto agglomerates would turn it into crack initiation sites [43]. In short, better dispersion contributed to more efficient stress transfer, eventually enhancing interfacial properties between the polymer and matrix, as well as mechanical properties. This was proven from the mechanical test conducted, which showed better dispersion leads to significantly higher tensile and elongation at break. In comparison with bionanocomposite with smaller or no agglomeration, one with large agglomeration experienced break at low elongation due to its inability to withstand increased stress.

**Figure 5:**
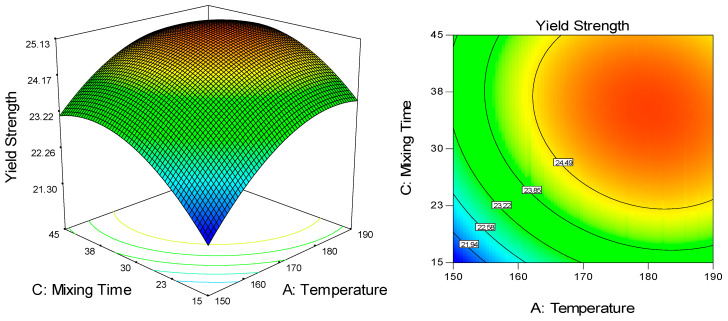
The 3D and contour plot for the dependence of UHMWPE/CNF bionanocomposite’s yield strength on temperature and mixing time as significant factors.

**Figure 6:**
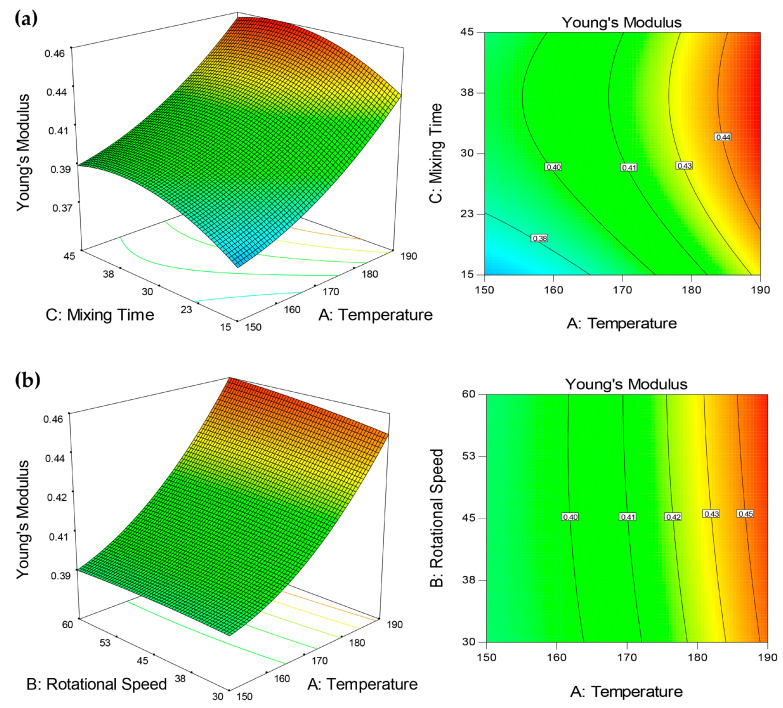
The 3D and contour plot for the dependence of UHMWPE/CNF bionanocomposite’s Young’s modulus on (**a**) temperature and mixing time, and (**b**) temperature and rotational speed as significant factors.

Similar to elongation at break, significant linear and quadratic effects of both temperature and mixing time were observed against yield strength and Young’s modulus with no significant interactions between all factors (Table 2). Increment of these responses can be seen in Figure 5 and Figure 6a, respectively, with increased temperature (150 °C to 190 °C) and mixing time (15 min to 45 min). It was aforementioned that high temperature improves chain mobility, resulting in higher cross-linked polymer chains and better mechanical strength [19]. However, different from tensile strength, the presence of agglomeration at a temperature higher than 170 °C did not negatively affect the yield strength and Young’s modulus. This can be explained by the fact that agglomeration causes the polymer to rupture during elongation, attributing to inefficient stress transfer [44,45,46,47,48,49]. In this stage, increased stress and strain led to plastic deformation beyond the yield point and occurrence of rupture, preventing the achievement of high tensile strength. In contrast, Young’s modulus indicates stiffness/linear elasticity of the material, portraying its ability to stand stress before undergoing deformation, while yield strength indicates the ability of the material to endure yielding related to plastic deformation [50,51].

**Figure 7:**
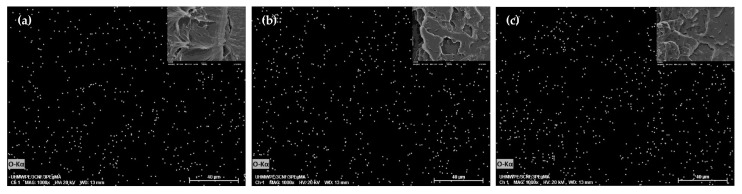
SEM-EDS images of UHMWPE/CNF bionanocomposites fabricated at a rotational speed of (**a**) 30 rpm, (**b**) 45 rpm, and (**c**) 60 rpm with constant temperature (170 °C) and mixing time (30 min).

Meanwhile, CNF dispersion and distribution in UHMWPE were found to be unaffected by rotational speed, as proved by insignificant changes of tensile strength, yield strength, and elongation. Yet, a linear significant effect was seen on Young’s modulus (Table 2), whereby slight improvement could be observed when UHMWPE bionanocomposite was processed at a higher rotational speed (Figure 6b). The rotational speed relates to the shear stress of polymer; increasing shear stress promotes polymer melt infiltration into filler agglomerates. This weakens agglomerates packing and structure, resulting in smaller agglomerates and better dispersion [52]. However, the effects of shear stress and melt infiltration on filler’s dispersion primarily depend on the melt viscosity of the polymer. A low rotational speed has been reported to provide better filler dispersion in high viscosity matrix, while there is an insignificant effect of rotational speed on low viscosity matrix [53]. In this study, rotational speed was found to not affecting CNF dispersion despite the high melt viscosity of UHMWPE. It is postulated that the use of triple screw kneading extruder during mixing assisted in breaking and mixing CNF aggregates, and hence the effect of rotational speed was less pronounced. This was proved through the SEM-EDS analysis, as shown in Figure 7.

### 2.3. Response Surface Optimization of UHMWPE/CNF Biocomposites

In accordance with the design and analysis conducted, numerical optimization was carried out by considering the criteria of each factor (Table 3). The tensile strength and yield strength were set within range due to the small difference between the lower range value and the upper range value of both responses. Besides, the highest tensile strength, which was 25.3 MPa, was obtained through increased temperature up to 170 °C, attributing to improved polymer chain mobility. However, at this particular temperature, the elongation at break reduced very dramatically by almost 80% as compared to a lower temperature of 150 °C, which was due to the appearance of agglomeration. Due to the severity effect on elongation at break, this response was set at a maximum value, as well as Young’s modulus, indicating the stiffness of the material. As shown in Table 3, the optimal temperature, rotational speed, and mixing time for UHMWPE/CNF bionanocomposite fabrication were 150 °C, 60 rpm, and 45 min, respectively. The predicted tensile strength, yield strength, elongation at break, and Young’s modulus for this optimum condition were 22.83 MPa, 23.14 MPa, 487.31%, and 0.391 GPa, respectively, with maximum desirability of 0.830.

### 2.4. Validation Experiment

**Figure 8:**
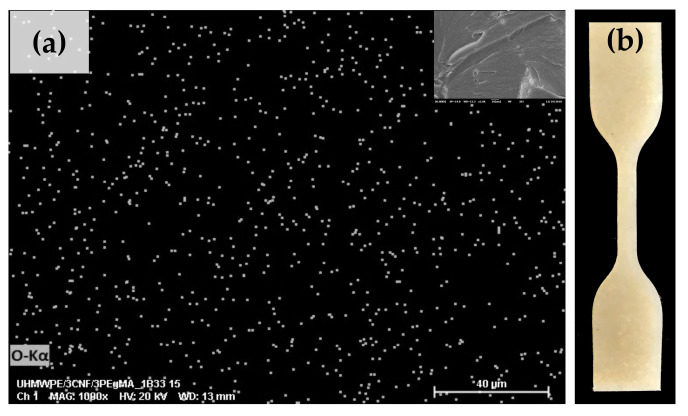
The (**a**) SEM-EDS and (**b**) visual images of UHMWPE/CNF bionanocomposites fabricated using an optimum processing condition.

Throughout the validation experiment, mechanical properties of UHMWPE/CNF bionanocomposite fabricated at the suggested parameter were in agreement with the predicted value, as proposed by the model (Table 4), except for tensile strength that was 22.8% higher. In consideration of the aim of this study, which was to achieve good CNF dispersion and high mechanical properties, this result was considerably good and favorable. The experimental value of tensile strength obtained (28 MPa) surpassed the standard specification of the fabricated form of UHMWPE for surgical implant ASTM F648-14 (27 MPa) [54]. Accordingly, the SEM-EDS image of the bionanocomposites fabricated at this optimum condition showed relatively good filler distribution, represented by the white dots (oxygen element detection), which were present in CNF (Figure 8a). This was supported by the visual appearance of the UHMWPE/CNF bionanocomposites, which exhibited homogenous filler distribution without the presence of agglomerated CNF (Figure 8b).

Meanwhile, the UHMWPE/CNF bionanocomposites (UHMWPE/3% CNF/3% MAPE) fabricated at this optimum condition exhibited 18% and 19% higher Young’s and flexural modulus, respectively, as compared to UHMWPE (Table 5). This finding proved the potential of CNF in improving the stiffness of UHMWPE for better abrasion and wear resistance as tibial inserts material [55,56]. Even so, the amount of filler loading should be identified and optimized in order to not defying the tensile and elongation properties of the UHMWPE/CNF bionanocomposites and will be reported in future work. It is also worth noting that yield strength is rather important and appropriate for evaluating bionanocomposites as compared to tensile strength, as it indicates the ability of materials to withstand load before deforming plastically. As such, once a material of composite implant yields under service conditions, it is considered a failure [57].

### 2.5. Effect of MAPE as Compatibilizer

**Figure 9:**
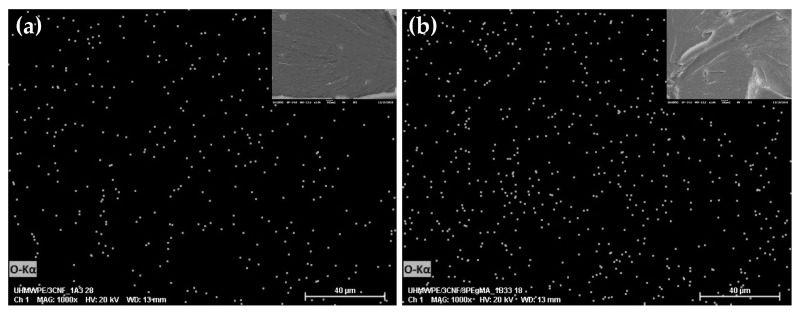
SEM-EDS images of (**a**) uncompatibilized and (**b**) MAPE (maleic anhydride-grafted-polyethylene)-compatibilized UHMWPE/CNF bionanocomposites.

Homogenous filler distribution in composites also depends on the matrix-filler compatibility. The addition of compatibilizers, such as MAPE, has been often used to improve compatibility between hydrophobic and hydrophilic polyethylene, eventually aiding in the improvement of mechanical properties [4,58,59]. Nevertheless, the effect of compatibilizer on ultra-high molecular weight polyethylene, in this study, was found to be insignificant in improving mechanical properties (Table 5). Through SEM-EDS image (Figure 9), even though the CNF distribution in MAPE–compatibilized bionanocomposites was seen to be slightly better than the uncompatibilized bionanocomposites, no agglomeration was observed in both images. This indicated that through the optimized bionanocomposites fabrication process, a good mixing, resulting in a relatively homogenous filler dispersion in the matrix, was successfully achieved, even without the presence of a compatibilizer.

By referring to the mechanical properties of UHMWPE/CNF bionanocomposites, as shown in Table 5, there was no significant difference in tensile strength, elongation at break, and flexural between uncompatibilzed and compatibilized UHMWPE/CNF bionanocomposites. The yield strength and Young’s modulus of uncompatibilized UHMWPE/CNF bionanocomposites were slightly but significantly higher. In this case, it suggested that MAPE incorporation was unnecessary for UHMWPE/CNF bionanocomposite fabrication.

Rather than strengthening and compatibilizing the UHMWPE and CNF, the low molecular weight MAPE in the bionanocomposites interfered with the highly dense and packed arrangement of UHMWPE linear chains [60,61]. As a result, the intermolecular forces binding of the polymer became weaker, and when polymer chains moved during deformation under stress, the presence of weak link and high cross-link in a polymer resulted in the non-equilibrium force acting on the weak link [62]. This caused molecular chain slippage, thus explaining the relatively lower yield strength and Young’s modulus as well as higher elongation. These properties represent the resistance of materials to plastic deformation, by which when the load increased beyond the yield point, the materials could no longer behave elastically but experienced permanent distortion, causing it to elongate and break.

## 3. Materials and Methods

### 3.1. Materials

Ultra-high molecular weight polyethylene (UHMWPE) was purchased from Sigma-Aldrich (St. Louis, MO, USA), in the form of fine powder with an average molecular weight of 3 × 10^6^–6 × 10^6^ g/mol. Maleic anhydride-grafted-polyethylene (MAPE) containing 0.5 wt.% maleic anhydride was purchased from the same manufacturer and was used in the experiment as compatibilizer. The melting point and density of UHMWPE and MAPE are 138 °C, 0.94 g/mL and 107 °C, 0.92 g/mL, respectively. Meanwhile, 2 wt.% cellulose nanofiber (CNF) in the slurry form was purchased from ZoepNano Sdn. Bhd., (Serdang, Malaysia), containing fiber with a diameter size of less than 50 nm.

### 3.2. Bionanocomposite Fabrication and Molding

**Figure 10:**
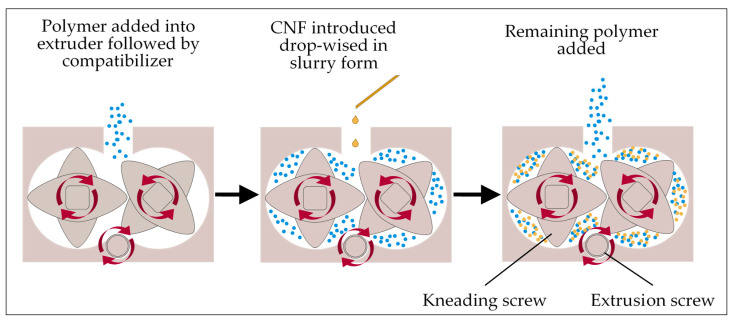
Preparation of UHMWPE/CNF bionanocomposite by using a triple screw kneading extruder.

UHMWPE/CNF bionanocomposite was produced by using a triple screw kneading extruder at Kyushu Institute of Technology, Fukuoka, Japan (Figure 10). About 50% of total UHMWPE resin was first introduced into the extruder, followed by 3 wt.% MAPE as a compatibilizer. A 3 wt.% CNF was later added into the mixture drop-wise before the rest of UHMWPE was added in. Afterward, the sample was molded into 10 cm × 10 cm film by direct compression molding at a temperature of 175 °C and 15 MPa pressure for 45 min [63].

### 3.3. Characterization of Bionanocomposite

#### 3.3.1. Determination of Mechanical Properties

Mechanical properties of the samples were analyzed using a compact tensile and compression tester IMC-18E0 (Imoto Machinery Co., Ltd., Kyoto, Japan). Tensile strength, yield strength, elongation at break, and Young’s modulus were performed on ASTM D638-02 tensile specimen at 50 mm min^−1^ crosshead speed, while flexural strength and flexural modulus were conducted at 10 mm min^−1^ speed (*n* = 8) [31,38]. The mechanical properties of fabricated bionanocomposites, after validation experiment, were analyzed statistically using one-way ANOVA and Duncan’s Multiple range test.

#### 3.3.2. Scanning Electron Microscopy-Energy-Dispersive Spectroscopy (SEM-EDS)

The CNF dispersion in UHMWPE/CNF bionanocomposite was analyzed by using a COXEM EM-30AX desktop scanning electron microscope (SEM) with integrated energy-dispersive spectroscopy (EDS) for elemental microanalysis (COXEM Co. Ltd., Daejeon, South Korea). Bionanocomposite samples were fractured in liquid nitrogen and sputter-coated with gold for 250 s (4 mA) prior to observation. The SEM-EDS micrographs were obtained with an acceleration voltage of 15 kV at 1000× magnification.

#### 3.3.3. Experimental Design and Optimization

The experiment was conducted according to a face-centered central composite design (CCD), with three varied factors—temperature (X_1_) (150 to 190 °C), rotational speed (X_2_) (30 to 60 rpm), and mixing time (X_3_) (15 to 45 min). Face-centered CCD with an alpha value 1.0 was used since the region of operability covered the full region of interest [64]. The temperature was set between 150 °C and 190 °C, in consideration of the melting point of UHMWPE from differential scanning calorimetry (DSC), which is approximately 140 °C, and onset degradation of CNF from thermogravimetric (TG) analysis at 210 °C. Three levels were selected for each factor, represented by coded units (−1), (0), and (+1), which are low, middle, and high level, respectively. Meanwhile, the mechanical properties of the tensile strength (Y_1_), yield strength (Y_2_), elongation-at-break (Y_3_), and Young’s modulus (Y_4_) were recorded as responses. Table 6 represents the experimental design, which consists of 20 runs, including six center points to reduce the variability in the data collection.

The results obtained were analyzed by using Design Expert statistical software (Version 7.0, Stat-Ease Inc., Minneapolis, MN, USA). The significance of each factor and the regression coefficient of linear, quadratic, and interaction terms were evaluated by considering the confidence level above 95% or *p*-value less than 0.05 using analysis of variance (ANOVA). A contour plot was used to show the effect of the factors on the responses and to locate the optimal levels. The predicted optimal condition obtained from software in terms of temperature, rotational speed, and mixing time was validated and verified by conducting an actual experiment. The system behavior was explained by the second-order polynomial equation, as shown in Equation (5), where Y_1_, Y_2_, Y_3_, and Y_4_ are the responses; X_1_, X_2_, and X_3_ are the varied factors ranging from -1 to 1, which influence the response Y; β_0_ is the constant coefficient; β_1_, β_2_, β_3_ are linear coefficients; β _11_, β_22_, β_33_ are quadratic coefficients; β _12_, β_13_, β_23_ are interaction coefficients. The validity and adequacy of the regression models were proved by comparing the experimental data obtained and the fitted value predicted by the models.

Y = β_0_ + β_1_X_1_ + β_2_X_2_ + β_3_X_3_ + β_11_X_1_^2^ + β_22_X_2_^2^ + β_33_X_3_^2^ + β_12_X_1_X_2_ + β_13_X_1_X_3_ + β_23_X_2_X_3_ (5)

## 4. Conclusions

Optimizing the melt blending processing condition of triple screw kneading extruder allowed homogenous CNF dispersion in the UHMWPE matrix despite the high melt viscosity of the polymer. At optimum temperature, rotational speed, and mixing time of 150 °C, 60 rpm, and 45 min, the CNF filler in UHMWPE polymer matrix was homogenously distributed, reporting the values of 22.83 MPa, 23.14 MPa, 487.31%, and 0.391 GPa for tensile strength, yield strength, elongation at break, and Young’s modulus, respectively. The incorporation of CNF increased the yield and flexural strength as well as Young’s and flexural modulus. Meanwhile, the effect of MAPE as a compatibilizer on the tensile, elongation at break, and flexural properties was found to be insignificant.

## Figures and Tables

**Table 1:** The experimental and predicted values of responses.

Run	Tensile Strength (MPa), Y_1_		Yield Strength (MPa), Y_2_		*** Ln Elongation (%), Ln Y_3_		Young’s Modulus (GPa), Y_4_	
	* Exp.	** Pred.	* Exp.	** Pred.	* Exp.	** Pred.	* Exp.	** Pred.
1	23.32	23.22	23.72	23.54	4.805	4.974	0.428	0.432
2	23.35	22.89	23.25	23.15	5.928	5.667	0.379	0.386
3	23.09	22.83	23.19	23.14	6.226	6.189	0.393	0.391
4	23.71	23.72	23.71	23.91	4.422	4.290	0.449	0.448
5	22.86	23.33	23.17	23.32	5.566	5.643	0.392	0.396
6	24.83	24.84	24.04	24.71	4.695	4.624	0.393	0.406
7	24.93	24.84	25.07	24.71	4.350	4.624	0.395	0.406
8	25.3	24.84	25.38	24.71	4.241	4.624	0.405	0.406
9	25.01	24.97	25.02	24.47	4.080	4.598	0.414	0.413
10	25.27	24.27	25.27	24.66	4.696	4.625	0.390	0.396
11	25.02	24.22	23.10	23.25	6.072	5.398	0.380	0.375
12	24.12	24.84	24.99	24.71	4.676	4.624	0.400	0.406
13	23.96	24.84	24.01	24.71	4.819	4.624	0.411	0.406
14	24.21	24.84	23.98	24.71	4.650	4.624	0.419	0.406
15	21.52	21.72	21.41	21.30	6.264	6.435	0.365	0.368
16	22.08	22.38	22.86	23.13	5.568	5.438	0.393	0.391
17	24.27	24.44	24.57	24.78	4.750	4.666	0.427	0.414
18	24.10	24.09	24.38	24.50	4.342	4.123	0.448	0.456
19	21.54	21.76	21.67	21.65	5.929	6.187	0.347	0.341
20	24.69	24.31	24.95	24.66	4.257	4.362	0.460	0.446

* Exp.: Experimental; ** Pred.: Predicted, *** Ln: Natural log.

**Table 2:** Analysis of variance (ANOVA) for response surface quadratic model.

	Tensile Strength (MPa), Y_1_	Yield Strength (MPa), Y_2_	Ln Elongation (%), Ln Y_3_	Young’s Modulus (GPa), Y_4_
Model	0.0069 *	0.0022 *	0.0018 *	0.0002 *
Linear				
X_1_—Temperature	0.0070 *	0.0017 *	0.0002 *	<0.0001 *
X_2_—Rotational speed	0.6962	0.7467	0.8609	0.0238 *
X_3_—Duration	0.1053	0.0065 *	0.0056 *	0.0003 *
Interaction				
X_1_X_2_	0.9430	0.4915	0.1017	0.5871
X_1_X_3_	0.8088	0.2922	0.2627	0.9362
X_2_X_3_	0.6241	0.6621	0.3470	0.0946
Quadratic				
X_1_^2^	0.0116 *	0.0390 *	0.1022	0.1563
X_2_^2^	0.2558	0.9787	0.9240	0.8894
X_3_^2^	0.5640	0.0313 *	0.1153	0.0863
Lack of fit	0.2230 **	0.7311 **	0.0726 **	0.7610 **
R^2^	0.8314	0.8685	0.8745	0.9258
Standard deviation	0.6642	0.5630	0.3599	0.0107

* statistically significant at *p* < 0.05 for model; ** statistically insignificant at *p* > 0.05 for the lack of fit test.

**Table 3:** The settings and solutions of the numerical optimization criterion.

Factors Constraints									
Name		Goal		Lower Limit			Upper Limit		
X_1_		is in range		150.00			190.00		
X_2_		is in range		30.00			60.00		
X_3_		is in range		15.00			45.00		
**Response Constraints**									
Y_1_		is in range		21.52			25.30		
Y_2_		is in range		21.41			25.38		
Y_3_		maximize		59.17			525.43		
Y_4_		maximize		0.347			0.460		
**Optimum Solutions**									
Number	X_1_		X_2_	X_3_	Y_1_	Y_2_	Y_3_	Y_4_	Desirability
1	150.00		60.00	45.00	22.83	23.14	487.31	0.391	0.830
2	150.00		59.88	45.00	22.83	23.14	485.61	0.391	0.829
3	150.00		59.45	45.00	22.85	23.14	479.90	0.391	0.825
4	150.67		60.00	45.00	22.93	23.22	458.69	0.391	0.811

**Table 4:** Comparison between predicted and experimental values of UHMWPE/CNF bionanocomposites fabricated at optimal conditions.

	Predicted	Experimental
Tensile strength (MPa), Y_1_	22.8	28.0 ± 1.9
Yield strength (MPa), Y_2_	23.1	22.8 ± 0.3
Elongation (%), Y_3_	487.3	461.6 ± 40.0
Young modulus (GPa), Y_4_	0.391	0.366 ± 0.018

**Table 5:** Mechanical properties of uncompatibilized and MAPE-compatibilized UHMWPE/CNF bionanocomposites (*p* < 0.05).

	UHMWPE	UHMWPE/ 3% CNF/ 0% MAPE	UHMWPE/ 3% CNF/ 3% MAPE
Tensile strength (MPa)	35.5 ± 2.7 ^a^	27 ± 1.9 ^b^	28 ± 1.9 ^b^
Yield strength (MPa)	23 ± 0.2 ^b^	24 ± 0.4 ^a^	23 ± 0.3 ^b^
Elongation (%)	669 ± 51.5 ^a^	432 ± 49.5 ^b^	462 ± 40.3 ^b^
Young’s modulus (MPa)	329 ± 5.2 ^b^	389 ± 15.3 ^a^	366 ± 17.7 ^b^
Flexural strength (MPa)	102 ± 21.5 ^b^	158 ± 23.8 ^a^	126 ± 15.5 ^a,b^
Flexural modulus (MPa)	175 ± 15.0 ^b^	216 ± 8.0 ^a^	208 ± 18.3 ^a^

UHMWPE: ultra high molecular weight polyethylene; CNF: cellulose nanofiber; MAPE: maleic anhydride-grafted-polyethylene. Letters a and b indicate significant difference based on statistical analysis (*p* < 0.5).

**Table 6:** Central composite design matrix of coded and actual factor level.

Run	Temperature (°C), X_1_		Rotational Speed (rpm), X_2_		Mixing Time (min), X_3_	
	Coded	Actual	Coded	Actual	Coded	Actual
1	+1	190	+1	60	−1	15
2	−1	150	0	45	0	30
3	−1	150	+1	60	+1	45
4	+1	190	−1	30	+1	45
5	+1	190	−1	30	−1	15
6	0	170	0	45	0	30
7	0	170	0	45	0	30
8	0	170	0	45	0	30
9	0	170	0	45	+1	45
10	0	170	−1	30	0	30
11	0	170	0	45	−1	15
12	0	170	0	45	0	30
13	0	170	0	45	0	30
14	0	170	0	45	0	30
15	−1	150	+1	60	−1	15
16	−1	150	−1	30	+1	45
17	0	170	+1	60	0	30
18	+1	190	+1	60	+1	45
19	−1	150	−1	30	−1	15
20	+1	190	0	45	0	30
